# MixTURE: *L*1-Norm-Based Mixed Second-Order Continuity in Strain Tensor Ultrasound Elastography

**DOI:** 10.1109/TUFFC.2024.3449815

**Published:** 2024-11-27

**Authors:** Md Ashikuzzaman, Arunima Sharma, Nethra Venkatayogi, Eniola Oluyemi, Kelly Myers, Emily Ambinder, Hassan Rivaz, Muyinatu A. Lediju Bell

**Affiliations:** Department of Electrical and Computer Engineering, Johns Hopkins University, Baltimore, MD 21218 USA; Department of Electrical and Computer Engineering, Johns Hopkins University, Baltimore, MD 21218 USA.; Department of Computer Science, Johns Hopkins University, Baltimore, MD 21218 USA.; Department of Radiology and Radiological Science, Johns Hopkins Medicine, Baltimore, MD 21287 USA.; Department of Radiology and Radiological Science, Johns Hopkins Medicine, Baltimore, MD 21287 USA.; Department of Radiology and Radiological Science, Johns Hopkins Medicine, Baltimore, MD 21287 USA.; Department of Electrical and Computer Engineering, Concordia University, Montreal, QC H3G 1M8, Canada; Department of Electrical and Computer Engineering, Department of Biomedical Engineering, Department of Computer Science, and Department of Oncology, Johns Hopkins University, Baltimore, MD 21218 USA

**Keywords:** Analytic optimization, mixed second-order derivative, regularized tracking, strain tensor imaging, ultrasound elastography

## Abstract

Energy-based displacement tracking of ultrasound images can be implemented by optimizing a cost function consisting of a data term, a mechanical congruency term, and first- and second-order continuity terms. This approach recently provided a promising solution to 2-D axial and lateral displacement tracking in ultrasound strain elastography. However, the associated second-order regularizer only considers the unmixed second derivatives and disregards the mixed derivatives, thereby providing suboptimal noise suppression and limiting possibilities for total strain tensor imaging. We propose to improve axial, lateral, axial shear, and lateral shear strain estimation quality by formulating and optimizing a novel L1-norm-based second-order regularizer that penalizes both mixed and unmixed displacement derivatives. We name the proposed technique L1-MixTURE, which stands for L1-norm Mixed derivative for Total UltRasound Elastography. When compared with simulated ground-truth results, the mean structural similarity (MSSIM) obtained with L1-MixTURE ranged 0.53–0.86 and the mean absolute error (MAE) ranged 0.00053–0.005. In addition, the mean elastographic signal-to-noise ratio (SNR) achieved with simulated, experimental phantom, and in vivo breast datasets ranged 1.87–52.98, and the mean elastographic contrast-to-noise ratio (CNR) ranged 7.40–24.53. When compared with a closely related existing technique that does not consider the mixed derivatives, L1-MixTURE generally outperformed the MSSIM, MAE, SNR, and CNR by up to 37.96%, 67.82%, and 25.53% in the simulated, experimental phantom, and in vivo datasets, respectively. These results collectively highlight the ability of L1-MixTURE to deliver highly accurate axial, lateral, axial shear, and lateral shear strain estimates and advance the state-of-the-art in elastography-guided diagnostic and interventional decisions.

## Graphical Abstract

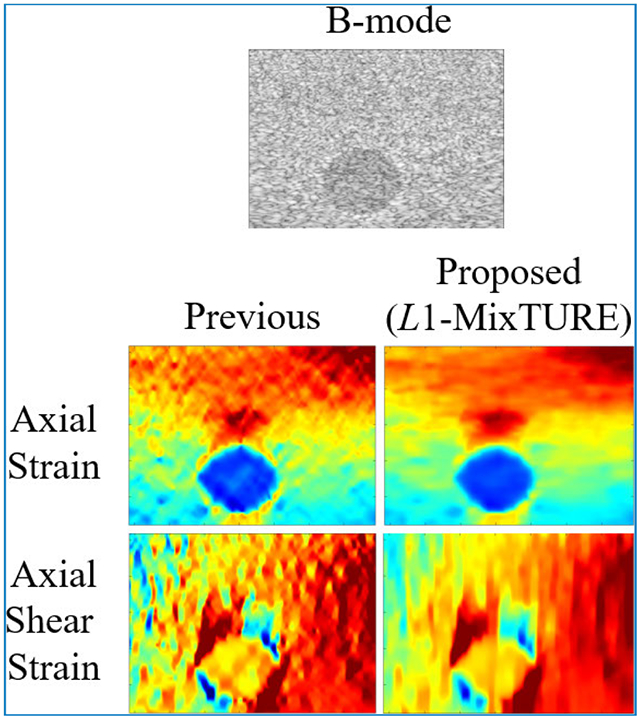

## I. Introduction

Ultrasound strain elastography (also known as ultrasound strain imaging) [1] refers to a noninvasive medical imaging technique that maps tissue strain as an indicator of tissue elasticity to distinguish between healthy and pathologic tissues. This distinction is based on the assumption that pathologic tissues (e.g., malignant tumors, benign lesions) have elastic characteristics that differ from healthy tissues [2]. Ultrasound strain elastography [3] has thus far been deployed in applications such as liver health monitoring [4], [5], [6], breast tissue characterization [7], [8], cardiovascular health assessment [9], [10], [11], ablation monitoring [12], [13], and muscle tissue examination [14], [15]. To implement this approach, time-series radio frequency (RF) ultrasound frames are acquired from the tissue of interest undergoing uniaxial compression with a handheld ultrasound probe. Two frames, typically containing an axially dominant motion pattern, are selected from the acquired RF frames. The displacement field between the selected RF frames is calculated using a displacement or motion-tracking algorithm (also known as speckle tracking). Finally, the estimated displacement components (i.e., axial and lateral) are spatially differentiated to obtain the strain maps.

As the quality of displacement tracking directly impacts the accuracy of the estimated strain, the incorporated displacement estimation algorithm plays a pivotal role in the success of a strain imaging framework. However, displacement tracking is a nontrivial task in ultrasound strain elastography, due to the ill-posed nature of ultrasound data (e.g., many samples with the same amplitude, characteristics, and similar patterns) are present in a single frame of ultrasound data) [16]. Despite this challenge, three broad classes of techniques, namely, window-based [8], [17], [18], [19], [20], [21], [22], [23], deep learning-based [24], [25], [26], [27], and energy-based [11], [16], [28], [29], [30], [31], [32], [33], are available to accomplish the critical task of displacement tracking [34].

Among the three general categories of displacement estimation algorithms, window-based techniques segment a precompressed frame into multiple overlapping windows and search for the contents of each window in the postcompressed frame to find the best match. The best match between the pre- and postcompressed window segments is typically determined by finding the location that corresponds to the maximum normalized cross correlation (NCC) [18], [19] or zero-phase-crossing [17]. Although window-based algorithms are generally known to be noise-sensitive, a partial differential equation-based postprocessing step was recently introduced [8], [35] to iteratively regularize displacement fields estimated by window-based tracking algorithms to reduce the noise that would otherwise be present. However, this noise reduction is achieved at the cost of high computation time due to the thousands of iterations required to achieve the desired result [8].

Deep-learning-based algorithms are comparatively new alternatives to estimate displacement fields in ultrasound elastography [25], [26], [36] with less time than that required with window-based techniques [16], [37]. In most cases, a large training dataset is required to obtain acceptable performance [24]. Recently proposed unsupervised training approaches [26], [27] have somewhat reduced the data hunger of deep-learning-based displacement estimation algorithms.

In contrast to window-based and deep-learning-based techniques, energy-based techniques (also known as techniques that require regularized optimization) solve the displacement tracking problem in two steps. The first step devises the problem as a regularized cost function, which is then optimized in the second step. The cost function is typically formulated considering data constancy (i.e., the similarity of RF data between the pre- and postcompressed frames), displacement continuity, and tissue mechanics-driven constraints to model the tissue deformation physics. Energy-based techniques are generally less sensitive to noise [16], [38] than window-based and deep-learning-based algorithms. In addition, the energy-based techniques do not require any training data.

Energy-based algorithms also address the ill-posed, underdetermined nature of displacement tracking by introducing displacement continuity constraints to the associated cost function. A weighted combination of the first- and second-order displacement derivatives [16] has shown promise in physics-based modeling of the continuity term in an elastographic cost function. In particular, second-order ultrasound elastography (SOUL) [16] considers the L2-norm of the second-order derivatives to construct the continuity constraint and render smooth strain maps, without losing the target-to-background contrast. However, the L2-norm-based regularization embedded in SOUL penalizes the displacement discontinuities regardless of the underlying elasticity distribution and, therefore, blurs the inclusion edges. L1-norm-based SOUL (L1-SOUL) [32] resolves this edge-blurring issue by implementing L1-norm-based regularization. However, like most strain imaging techniques, SOUL and L1-SOUL focus on axial strain imaging and are not suitable for high-quality lateral strain imaging.

The limitation of low-quality lateral strain imaging was mitigated by mechanically inspired SOUL (MechSOUL) [38] and mechanically inspired L1-SOUL (L1-MechSOUL) [38]. L1-MechSOUL leverages the integrated relationships driven by the effective Poisson’s ratio between axial and lateral strains, data similarity, and L1-norm-based displacement continuity constraints to produce sharper and more accurate axial and lateral strain images than MechSOUL. Similar to SOUL, L1-SOUL, and MechSOUL, L1-MechSOUL uses only the unmixed second derivatives $\left[\partial_{y}^{2}(\cdot ),\partial_{x}^{2}(\cdot )\right]$ to devise the second-order displacement regularizer. This simplified regularization scheme that does not consider the mixed second derivatives $\left[\partial_{xy}^{2}(\cdot )\right]$ regularizes the strain components (i.e., axial, lateral, axial shear, and lateral shear) in certain directions only, disregarding the others. This selective continuity framework fails to leverage the full potential of second-order regularization and, therefore, is a barrier to high-accuracy estimation of the total strain tensor (axial, lateral, axial shear, and lateral shear). Previous work addresses this concern using the mixed second derivative in an L2-norm-based optical flow framework designed for vascular elastography [11]. However, as noted above and in previous publications [32], [39], the L2-norm often overpenalizes displacement and strain discontinuities, resulting in blurred elastic property boundaries, whereas an L1-norm-based regularization framework generally provides sharper boundaries [32], [38].

With respect to breast mass imaging, traditional B-mode ultrasound imaging can be challenged by poor contrast between the mass and its surrounding tissue [40], [41], [42], [43]. In such low-contrast scenarios, strain images can complement B-mode ultrasound images to more reliably distinguish lesion boundaries relative to healthy tissue [3], [44]. In addition, the strain contrast between a breast mass and surrounding tissue can potentially serve as a biomarker of malignancy [45], [46]. Similarly, the bonding between the tumor and the surrounding tissue (which can be obtained by analyzing the shear strain at tumor edges) can also be a promising biomarker of tumor malignancy [47], [48]. As a potential treatment monitoring technique, strain imaging can be used to assess the response of a tumor to chemotherapy and other neoadjuvant or adjunct therapies [49], [50], [51], [52]. In these multiple diagnostic and interventional scenarios, the utility of strain imaging relies on high-accuracy, low-variance estimations of tissue displacement and strain. An erroneous and noisy strain estimate can lead to suboptimal sensitivity [53], an incorrect clinical inference, irrevocable interventional error, or morbid (possibly fatal) patient prognosis. Therefore, accurate, noise-free, edge-preserving strain estimates are paramount to the clinical success of ultrasound strain elastography.

Herein, we aim to improve accuracy and suppress noise within L1-MechSOUL axial, lateral, and shear strain images by formulating a novel L1-norm-based (alternatively known as “total variation”) second-order regularizer containing both mixed and unmixed derivatives. While improving axial and lateral strains [8], [38] is a common approach to improve ultrasound strain estimates, our approach to improve the shear strain estimates is additionally motivated by its potential to improve breast mass diagnosis [47], [48], [54]. We name the proposed algorithm L1-norm Mixed derivative for Total UltRasound Elastography (abbreviated as L1-MixTURE). L1-MixTURE uses the mixed second derivative in an L1-norm-based generalized strain tensor imaging framework that improves strain tracking in the axial, lateral, and shear directions. In addition, the work presented herein extends our associated conference paper [55] by presenting more details on methods and materials, validating with additional and more challenging in silico, phantom, and in vivo breast datasets, reporting axial, lateral, axial shear, and lateral shear strain components (rather than only axial and lateral strain components), and presenting comprehensive results and analyses.

## II. Methods

### A. Proposed Algorithm

#### 1) L1-MechSOUL:

The proposed L1-MixTURE algorithm is derived from L1-MechSOUL [38], which both require two RF frames collected before and after deforming a tissue. We refer to these two frames as $I_{1}(i,j)$ and $I_{2}(i,j)$, where 1≤i≤m and 1≤j≤n. Our goal is to first estimate the displacement fields between $I_{1}$ and $I_{2}$, and then spatially differentiate the displacement fields to calculate the strain components experienced by the interrogated tissue. To achieve this goal, the gross axial and lateral displacement estimates, a and l, respectively, are first estimated using dynamic programming [56]. Then, the initial displacement fields are refined using energy-based speckle tracking algorithms (e.g., L1-MechSOUL, L1-MixTURE) that optimize nonlinear cost functions in a continuous manner.

To implement L1-MechSOUL, we formulate a regularized nonlinear cost function $C_{l1m}$ comprising L2-norm data fidelity term, L1-norm first and second-order continuity terms, and L1-norm mechanical constancy constraint, as follows:

(1) $$C_{l1m}\left(\Delta a_{1,1},\ldots ,\Delta a_{m,n},\Delta l_{1,1},\ldots ,\Delta l_{m,n}\right)\;={\left\|D_{I}\left(i,j,a_{i,j},l_{i,j},\Delta a_{i,j},\Delta l_{i,j}\right)\right\|}_{2}^{2}+R_{1}+R_{2}+R_{m}$$

where Δa and Δl indicate incremental displacement fields, and $D_{I}(\cdot )$, $R_{1},\;R_{2}$, and $R_{m}$, respectively, denote the data term, first- and second-order continuity constraints, and the mechanical constancy term, defined as follows:

(2) $$D_{I}\left(i,j,a_{i,j},l_{i,j},\Delta a_{i,j},\Delta l_{i,j}\right)\;={\left[I_{1}(i,j)-I_{2}\left(i+a_{i,j}+\Delta a_{i,j},j+l_{i,j}+\Delta l_{i,j}\right)\right]}^{2}$$

(3) $$R_{1}=w_{f}\alpha_{1s}{\left\|\partial_{y}a-\epsilon_{aa}\right\|}_{1}+w_{f}\alpha_{2s}{\left\|\partial_{x}a-\epsilon_{al}\right\|}_{1}+w_{f}\beta_{1s}{\left\|\partial_{y}l-\epsilon_{la}\right\|}_{1}+w_{f}\beta_{2s}{\left\|\partial_{x}l-\epsilon_{ll}\right\|}_{1}$$

(4) $$R_{2}=w_{s}\alpha_{1s}{\left\|\partial_{y}^{2}a\right\|}_{1}+w_{s}\alpha_{2s}{\left\|\partial_{x}^{2}a\right\|}_{1}+w_{s}\beta_{1s}{\left\|\partial_{y}^{2}l\right\|}_{1}+w_{s}\beta_{2s}{\left\|\partial_{x}^{2}l\right\|}_{1}$$

(5) $$R_{m}=\alpha_{3s}{\left\|{\left(\partial_{x}l\right)}_{i,j}+v_{i,j}{\left(\partial_{y}a\right)}_{i,j}\right\|}_{1}$$

where $\partial_{y}(\cdot )$ and $\partial_{x}(\cdot )$ refer to the first-order axial and lateral derivatives, respectively, $w_{f},\;\alpha_{1s},\;\alpha_{2s},\;\beta_{1s}$, and $\beta_{2s}$ are the first-order regularization weights, $\epsilon_{aa},\;\epsilon_{al},\;\epsilon_{la}$, and $\epsilon_{\mathrm{ll}}$ contain the adaptive regularization parameters [16], [38], $\partial_{y}^{2}(\cdot )$ and $\partial_{x}^{2}(\cdot )$ denote unmixed second-order axial and lateral derivatives, respectively, $w_{s}$ controls the strength of second-order regularization, $\alpha_{3s}$ is the mechanical constancy controlling parameter, and $v_{i,j}$ is the effective Poisson’s ratio, which is defined as the negative of the samplewise ratio of lateral and axial strains, initiated with the target-specific nominal value and updated iteratively. As described in [38], L1-MechSOUL analytically optimizes $C_{l1m}$ in an iterative manner to determine the displacement fields.

#### 2) L1-MixTURE:

L1-MixTURE can be considered as a derivative of L1-MechSOUL by first recognizing that the L1-MechSOUL cost function, $C_{l1m}$, in (1) only considers the unmixed second derivatives, $\partial_{y}^{2}(\cdot )$ and $\partial_{x}^{2}(\cdot )$, and disregards the mixed second-order derivatives. However, the mixed derivatives contain specific smoothness features that are not fully used when only considering the unmixed second derivatives.

To provide an intuitive realization of second-order displacement derivatives, the unmixed second-order derivatives of the axial and lateral displacement fields are defined as follows:

(6) $$\partial_{y}^{2}(a)=\partial_{y}\left(\partial_{y}(a)\right)=\partial_{y}(\text{axial}\;\text{strain})$$

(7) $$\partial_{x}^{2}(a)=\partial_{x}\left(\partial_{x}(a)\right)=\partial_{x}(\text{axial}\;\text{shear}\;\text{strain})$$

(8) $$\partial_{y}^{2}(l)=\partial_{y}\left(\partial_{y}(l)\right)=\partial_{y}(\text{lateral}\;\text{shear}\;\text{strain})$$

(9) $$\partial_{x}^{2}(l)=\partial_{x}\left(\partial_{x}(l)\right)=\partial_{x}(\text{lateral}\;\text{strain})$$

where $\partial_{y}\left(a\right)=\text{axial}\;\text{strain},\;\partial_{x}\left(a\right)=\text{axial}\;\text{shear}\;\text{strain},\;\partial_{y}\left(l\right)=\text{lateral}\;\text{shear}\;\text{strain}$, and $\partial_{x}\left(l\right)=\text{lateral}\;\text{strain}$. Based on the definitions in (6)–(9), penalizing the unmixed derivatives regularizes the axial and lateral strains in the same direction as the respective strains, whereas the axial and lateral shear strains are regularized in the orthogonal direction to the respective shear strains. Therefore, the orthogonal directions of the axial and lateral strains and the direction of the axial and lateral shear strains remain unregularized. The mixed second-order derivatives fill these regularization gaps as follows:

(10) $$\partial_{xy}^{2}(a)=\partial_{x}\left(\partial_{y}(a)\right)=\partial_{x}(\text{axial}\;\text{strain})$$

(11) $$=\partial_{y}\left(\partial_{x}(a)\right)=\partial_{y}(\text{axial}\;\text{shear}\;\text{strain})$$

(12) $$\partial_{xy}^{2}(l)=\partial_{x}\left(\partial_{y}(l)\right)=\partial_{x}(\text{lateral}\;\text{shear}\;\text{strain})$$

(13) $$=\partial_{y}\left(\partial_{x}(l)\right)=\partial_{y}(\text{lateral}\;\text{strain}).$$

**Fig. 1.**
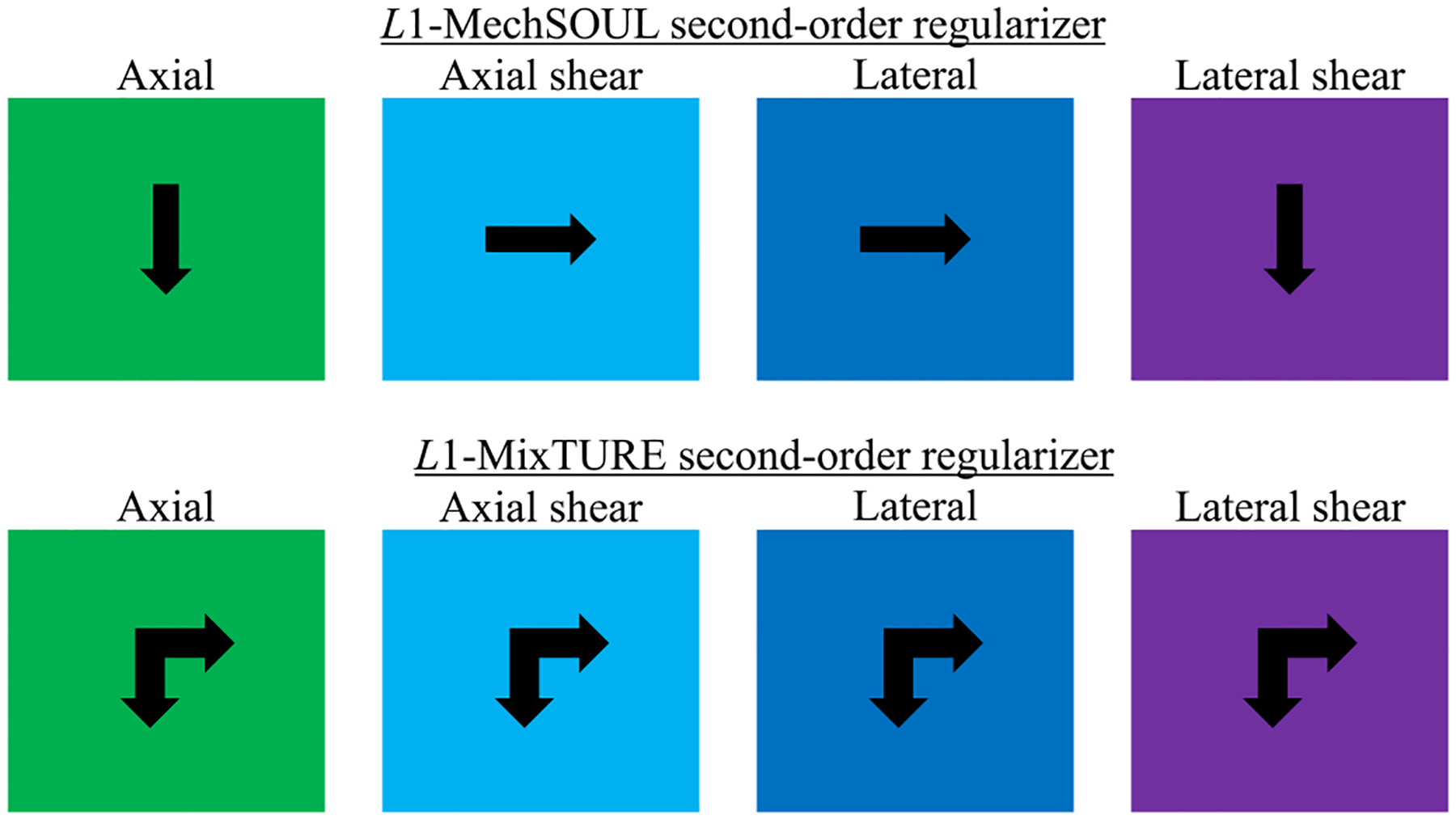
Difference between the L1-MechSOUL and L1-MixTURE second-order regularizers. An arrow indicates the direction of regularization.

Intuitively, the expressions in (10) and (11) indicate that the mixed second derivative of the axial displacement simultaneously regularizes both the axial strain in the lateral direction and the axial shear strain in the axial direction. Similarly, (12) and (13) indicate that the mixed second derivative of the lateral displacement axially regularizes the lateral strain and laterally regularizes the lateral shear strain. Therefore, combining the mixed and unmixed derivatives in a cost function can render a complete second-order regularization scheme and provide more accurate modeling of the elastographic motion, as illustrated in Fig. 1.

Based on the intuitive explanations provided above, L1-MixTURE considers both mixed and unmixed second-order displacement derivatives to construct the following cost function:

(14) $$C_{l1\text{mix}}\left(\Delta a_{1,1},\ldots ,\Delta a_{m,n},\Delta l_{1,1},\ldots ,\Delta l_{m,n}\right)\;=C_{l1m}\left(\Delta a_{1,1},\ldots ,\Delta a_{m,n},\Delta l_{1,1},\ldots ,\Delta l_{m,n}\right)+w_{s}\alpha_{\text{mix}}{\left\|\partial_{xy}^{2}a\right\|}_{1}+w_{s}\beta_{\text{mix}}{\left\|\partial_{xy}^{2}l\right\|}_{1}$$

where $\alpha_{\text{mix}}$ and $\beta_{\text{mix}}$ are the regularization weights of the mixed second derivatives, which are defined in terms of the displacements below

(15) $${\left(\partial_{xy}^{2}a\right)}_{i,j}=a_{i,j}+\Delta a_{i,j}-a_{i,j-1}-\Delta a_{i,j-1}-a_{i-1,j}-\Delta a_{i-1,j}+a_{i-1,j-1}+\Delta a_{i-1,j-1}$$

(16) $${\left(\partial_{xy}^{2}l\right)}_{i,j}=l_{i,j}+\Delta l_{i,j}-l_{i,j-1}-\Delta l_{i,j-1}-l_{i-1,j}-\Delta l_{i-1,j}+l_{i-1,j-1}+\Delta l_{i-1,j-1}.$$

Two simplification steps were performed before optimizing the cost function in (14). First, the first-order Taylor series expansion was performed on $I_{2}(\cdot )$ to remove the nonlinearity [i.e., the presence of $\Delta a_{i,j}$ and $\Delta l_{i,j}$ in $I_{2}(\cdot )$] from the data term defined in (2)

(17) $$I_{2}\left(i+a_{i,j}+\Delta a_{i,j},j+l_{i,j}+\Delta l_{i,j}\right)\approx I_{2}\left(i+a_{i,j},j+l_{i,j}\right)+\Delta a_{i,j}I_{2,a}^{\prime }+\Delta l_{i,j}I_{2,l}^{\prime }$$

where $I_{2,a}^{\prime }$ and $I_{2,l}^{\prime }$ denote the derivatives of $I_{2}$ in the axial and lateral directions, respectively. Second, the L1-norm was approximated with its differentiable version similar to [32] and [38]. The resulting simplified cost function, $C_{l1\text{mix},s}$, was then differentiated and iteratively optimized by setting the differential to zero (i.e., $\left(\left(\partial C_{l1\text{mix},s}(\cdot )\right)/\left(\partial \Delta a_{i,j}\right)\right)=0$ and $\left.\left(\left(\partial C_{l1\text{mix},s}(\cdot )\right)/\left(\partial \Delta l_{i,j}\right)\right)=0\right)$ and solving the resulting equation, leading to

(18) $$\left(H+D_{1}+\right.\left.D_{2,u}+D_{2,\text{mix}}+M\right)\Delta d\;=H_{1}\mu -\left(D_{1}+D_{2,u}+D_{2,\text{mix}}+M\right)d+b_{s}$$

where $H\in R^{2\text{mn}\times 2\text{mn}}$ and $H_{1}\in R^{2\text{mn}\times 2\text{mn}}$ are the symmetric tridiagonal and diagonal matrices, respectively, containing the derivatives of $I_{2}$ originating from the Taylor series expansion of (17) and the associated functions (e.g., squares of the axial and lateral derivatives, multiplications of axial and lateral derivatives), $D_{1},\;D_{2,u}$, and $D_{2,\text{mix}}$ are 2mn×2mn sparse matrices containing functions of first-order, unmixed second-order, and mixed second-order continuity weights, respectively, $M\in R^{2\text{mn}\times 2\text{mn}}$ is a matrix that comprises the functions of the effective Poisson’s ratio and the mechanical constancy weights, $d\in R^{2\text{mn}\times 1}$ is a vector containing the initial guesses provided by dynamic programming (i.e., a ad l) [56], $\Delta d\in R^{2\text{mn}\times 1}$ is a vector containing the incremental displacements (i.e., Δa and Δl), $\mu \in R^{2\text{mn}\times 1}$ is a vector that consists of the data residuals (i.e., the samplewise differences between $I_{1}$ and $I_{2}$ warped with a and l), and $b_{s}\in R^{2\text{mn}\times 1}$ is a vector containing functions of the adaptive regularization parameters and the first-order continuity weights.

After estimating Δd in (18), the incremental displacement fields, Δa and Δl, were added to the initial displacement fields, a and l, to obtain the final displacement estimates. These final axial and lateral displacement estimates were then spatially differentiated in the axial and lateral directions, respectively, to obtain the axial and lateral strains, respectively. The lateral spatial derivative of axial displacement provides the axial shear strain, whereas the axial spatial derivative of the lateral displacement generates the lateral shear strain. The spatial derivative operations were performed using a least-squares technique. While it is common to use a kernel size of 51 samples or more to implement the least-squares differentiator [32] and mask the outlier displacement samples, a large differentiation kernel smooths the strain image in a nondata-driven manner [32]. To avoid any nondata-driven smoothing of the strain estimates, the differentiation kernel size was set to three samples.

### B. Simulated Data

Four datasets were simulated prior to implementing the proposed L1-MixTURE algorithm. The first and second simulated datasets were created to qualitatively assess the performance of L1-MixTURE under multiple compression levels and with an inhomogeneous Poisson’s ratio, respectively. The third and fourth simulated datasets were included to quantitatively assess accuracy and image quality. The minimum simulated target size was 1.8 mm. For each simulation described below, the proposed L1-MixTURE algorithm described in Section II-A2 was implemented on selected pairs of beamformed RF data simulated by Field II [57] software.

The first simulated phantom contained a single slice of a 1.8-mm-thin layer as the target, designed to consider pathologies that extend beyond the lateral field of view of a transducer or targets that may naturally appear with thin, layered, or tubular shapes in ultrasound images (e.g., dense stromal fibrosis [40], cancer proliferating in an uncontrolled manner [58], possibly muscle [59], [60], myofascia [15], [37], [61], [62], nerve [63], [64], [65], vessel [66], [67], bone [68], or needle [69], [70] imaging as well). The background and target Young’s moduli were set to 10 and 20 kPa, respectively, whereas Poisson’s ratio of both target and background was set to 0.49. The phantom was uniaxially deformed with compression levels of 0.5%, 2%, 5%, and 8% using the closed-form equations described in the Supplementary Material of [6]. The resulting displacement fields were used to displace the precompression scatterer locations and obtain the postcompressed scatterer locations for each compression level. The pre- and postcompressed scatterer locations and the associated amplitudes were input to Field II [57] to simulate beamformed RF data. A 256-element transducer was modeled with 0.21-mm element width, 5-mm element height, 64 active elements, 60% fractional bandwidth, 7.27-MHz transmit center frequency, and 40-MHz sampling frequency. These parameters were selected to match those used in previous elastography research [38].

The second simulated phantom contained a cylindrical target with 10-mm face diameter, with target and background Poisson’s ratios of 0.25 and 0.45, respectively, which is supported by a previous work demonstrating that pathologic tissue may possess a different Poisson’s ratio than the surrounding healthy tissue [71]. Although Poisson’s ratio was inhomogeneous, the target had the same Young’s modulus (20 kPa) as the background. This phantom was uniaxially compressed by 2% using ABAQUS software (Providence, RI, USA). The pre- and postcompressed beamformed RF frames were simulated with Field II [57] using the same procedure and imaging parameters described above.

The third simulated dataset was a publicly available single-inclusion phantom [72] with background and target elastic moduli of 20 and 40 kPa, respectively. Poisson’s ratio was set to 0.49. This simulated phantom was deformed with uniaxial compression levels ranging from 0% (no motion) to 4.5% in increments of 0.5% using ABAQUS software (Providence, RI, USA). The corresponding pre- and post-compression beamformed RF data were simulated with Field II [57], with ultrasound transmit and temporal sampling frequencies of 5 and 50 MHz, respectively. White Gaussian noise was added to the beamformed RF data, resulting in 4.42-dB signal-to-noise ratio (SNR) and 20-dB peak SNR (PSNR), relative to the noiseless beamformed RF data.

The fourth simulated phantom (hereafter referred to as the “multi-inclusion phantom”) contained three stiff targets (elastic moduli: 40, 60, and 80 kPa) surrounded by a homogeneous background (elasticity: 20 kPa). Poisson’s ratio was 0.49. The phantom was compressed by 2% with a surface traction force (containing axial and lateral components) using ABAQUS. We simulated the pre- and postcompressed beamformed RF frames with Field II [57] using the same procedure and ultrasound imaging parameters as the first and second simulated phantoms. No electronic noise was added to this phantom, because the multi-inclusion simulated phantom is already challenged by having three inclusions of varying elasticities undergoing a surface traction force with axial and lateral components.

### C. Experimental Phantom Data

An Alpinion E-Cube 12R research ultrasound scanner connected to a 128-element L3–8 probe was used to conduct two beamformed RF data acquisitions from a CIRS breast elastography phantom (Model 059) at Johns Hopkins University. The imaging field of views of the first and second acquisitions contained one inclusion and two inclusions, respectively. The system was programmed with a transmit center frequency of 8 MHz, a sampling frequency of 40 MHz, and a transmit focus of 2 cm. The background elasticity of the phantom is 20±5kPa, whereas the elasticity of the inclusion is at least twice that of the background, as specified by the manufacturer. The first and second acquisitions consisted of 142 and 420 frames, respectively, as the phantom was axially compressed with the handheld ultrasound probe. Two consecutive frames of beamformed RF data belonging to each acquisition were used to create a reference precompressed B-mode image and to implement the proposed L1-MixTURE algorithm described in Section II-A2.

### D. In Vivo Breast Data

An Alpinion E-Cube 12R research ultrasound scanner connected to a 128-element L8–17 probe with a sampling frequency of 40 MHz, a transmit center frequency of 12 MHz, a transmit focus of 1.2 cm, and a 64-element receive aperture was used to acquire in vivo raw RF channel data. A patient scheduled for an ultrasound-guided core-needle biopsy of a suspicious breast mass was imaged prior to biopsy, after obtaining informed consent with approval from the Johns Hopkins Medicine Institutional Review Board (Protocol No. IRB00127110). With the patient in the supine position, the breast and underlying mass were axially compressed using the handheld ultrasound probe to simultaneously acquire 309 consecutive frames of channel data. Two consecutive frames of RF channel data were beamformed offline to create delay-and-sum beamformed RF data. These beamformed RF data were used to create a reference precompressed B-mode image and to implement the proposed L1-MixTURE algorithm described in Section II-A2. Pathology results indicated that a benign solid breast mass was imaged (i.e., a benign mass with stromal fibrosis and focal fibroadenomatoid changes).

### E. Benchmarking and Associated Parameter Selection

Two techniques were implemented to benchmark the performance of L1-MixTURE. The first technique is L1-MechSOUL [38], given its overlapping similarities to L1-MixTURE, as detailed in Section II-A2. The second technique is partial differential equation-refined window-based tracking [8] (hereafter referred to as PDE). This technique first incorporates an NCC-based window-matching and tracking algorithm to estimate the axial and lateral displacement fields. These axial and lateral displacement fields are then iteratively refined using a partial differential equation-based regularizer. PDE was chosen as a comparative technique for two reasons. First, PDE presents less-noisy versions of the displacement fields obtained by a standard window-based algorithm. Second, PDE is suitable for both axial and lateral strain imaging [8], [38].

To select optimal parameters when implementing L1-MixTURE, L1-MechSOUL, and PDE, a previously reported cross-validation procedure was implemented [38], which involved selecting validation beamformed RF images, and then tuning a range of parameters on the selected validation images. This parameter optimization was performed using one validation pair of beamformed RF frames per dataset type (i.e., simulated, phantom, and in vivo). The simulated validation images were selected from the public single-inclusion phantom data described in Section II-B, defined as 1% compression between pre- and postcompressed beamformed RF image frames. The experimental phantom validation images were selected from the multi-inclusion experimental acquisitions (see Section II-C), and the in vivo breast data validation images were selected from the associated data described in Section II-D. In each case, the selected experimental and in vivo validation images met the criteria of two beamformed RF images per dataset type, each with visually apparent uniaxial compression between selected image frames in the corresponding B-mode images. NCC-based window matching and tracking, L1-MechSOUL, and L1-MixTURE were then applied to the selected validation image pairs. The resulting L1-MechSOUL and L1-MixTURE parameter sets are reported in Table I. The selected parameter values are specific to the dataset type as different tissues have different quantitative and mechanical properties and exhibit varying noise characteristics. This approach has the potential to store organ-specific speckle-tracking parameters when implemented on commercial ultrasound scanners (similar to the parameter presets ultrasound machines use for B-mode imaging of different organs).

To reduce false peaks with NCC-based tracking during parameter tuning and subsequent benchmarking, the selected beamformed RF images were upsampled in the axial and lateral directions by a factor of 3 with a bicubic interpolation. A factor of 3 was selected to maintain a balance between the strain estimation quality and computational load (based on validation image results). The associated NCC-based tracking window size and overlap were selected as 15λ (i.e., 3×5λ) and 86%, respectively, to balance the required runtime with background smoothness, edge clarity, and target-to-background contrast. The resulting displacement fields were then resized to the original size of the input frames. The ratio of axial to lateral fidelity weights was 100 for the PDE-based refinement stage, as suggested in the original publication [8]. No upsampling of the input data was performed prior to using the energy-based displacement tracking techniques (i.e., L1-MechSOUL and L1-MixTURE), as these techniques are less sensitive to signal decorrelation.

The optimal parameters described above were applied to pairs of test images from each dataset. In particular, image test pairs with 0.5%, 2%, 5%, and 8% compressions between pre- and postcompressed beamformed RF image frames were selected from the thin-layer simulated phantom data described in Section II-B. For the single-inclusion simulated test data, beamformed RF image pairs with 2% compression between pre- and postcompressed beamformed data were selected from the associated data described in Section II-B. For the multi-inclusion and inhomogeneous Poisson’s ratio simulated test data, image pairs with 2% compression between pre- and postcompressed beamformed RF image frames were selected from the associated data described in Section II-B. For the experimental phantom and in vivo breast data, beamformed RF image pairs with visually apparent uniaxial compression in the corresponding B-mode images were selected as the test image pairs (i.e., the consecutive frame pairs described in Sections II-C and II-D). Each image in the test pair differed from the images selected for parameter optimization, and the resulting strain images obtained with the test data were used to assess and benchmark algorithm performance.

### F. Performance Metrics

In addition to qualitative evaluation, the mean structural similarity (MSSIM) and mean absolute error (MAE) between the estimated and ground-truth strain images were provided as strain estimation accuracy measurement metrics. MSSIM is defined in [73], and MAE is defined as follows:

(19) $$\text{MAE}=\frac{\sum_{j=1}^{n}\sum_{i=1}^{m}\left|{\overset{ˆ}{s}}_{i,j}-s_{i,j}\right|}{\text{mn}}$$

where ${\overset{ˆ}{s}}_{i,j}$ and $s_{i,j}$ are the estimated and ground-truth strains, respectively, for the pixel values located at index (i,j), with m and n total samples, respectively.

Elastographic SNR [2], [74] and contrast-to-noise ratio (CNR) [2] were additionally included as image quality assessment metrics

(20) $$\text{SNR}=\frac{\bar{s_{b}}}{\sigma_{b}}$$

(21) $$\text{CNR}=\sqrt{\frac{2{\left(\bar{s_{b}}-\bar{s_{t}}\right)}^{2}}{\sigma_{b}^{2}+\sigma_{t}^{2}}}$$

where $\bar{s_{b}}$ and $\bar{s_{t}}$ are the mean strain values within background and target regions of interest (ROIs), respectively, and $\sigma_{b}$ and $\sigma_{t}$ are the standard deviations of strain values within the same background and target ROIs, respectively. The CNR of the axial shear and lateral shear strains is not reported because the target-to-background contrast of the shear strain is not physically meaningful in most quasi-static elastography cases (although the strain distribution around target boundaries can portray the extent of bonding, which can help with tumor characterization [47]).

We use four metrics (i.e., MAE, MSSIM, SNR, and CNR) to summarize the accuracy of displacement and strain estimates and to assess image quality. Quantifying the displacement and strain estimation accuracy is important because it may provide correct information for tissue elastic properties, which enables better pathology detection. Instead of a single SNR or CNR value per dataset, as is common practice in previous elastography performance reports [17], a total of 30 SNR background ROIs (i.e., 30 3 × 3 mm ROIs) and 120 CNR ROI combinations (i.e., four 3 × 3 mm target ROIs combined with the same 30 background ROIs used to calculate SNR) were evaluated per dataset to better assess the overall image quality. The selected ROIs contained visually uniform background and target strains. The center of the background ROIs spanned 1.4–1.8-cm, 1.3–2.8-cm, 1.1–3-cm, 1.2–3.2-cm, and 0.8–1.7-cm axial distances and 1.5–2.4-cm, 0.6–2.2-cm, 0.67–3.5-cm, 2–3.7-cm, and 0.42–2.15-cm lateral distances in the single-inclusion simulated data, multi-inclusion simulated data, single-inclusion experimental phantom data, multi-inclusion experimental phantom data, and in vivo data, respectively. All the data processing and analyses were performed with MATLAB software (Natick, MA, USA).

## III. Results

### A. Validation With Multiple Compression Levels

**Fig. 2.**
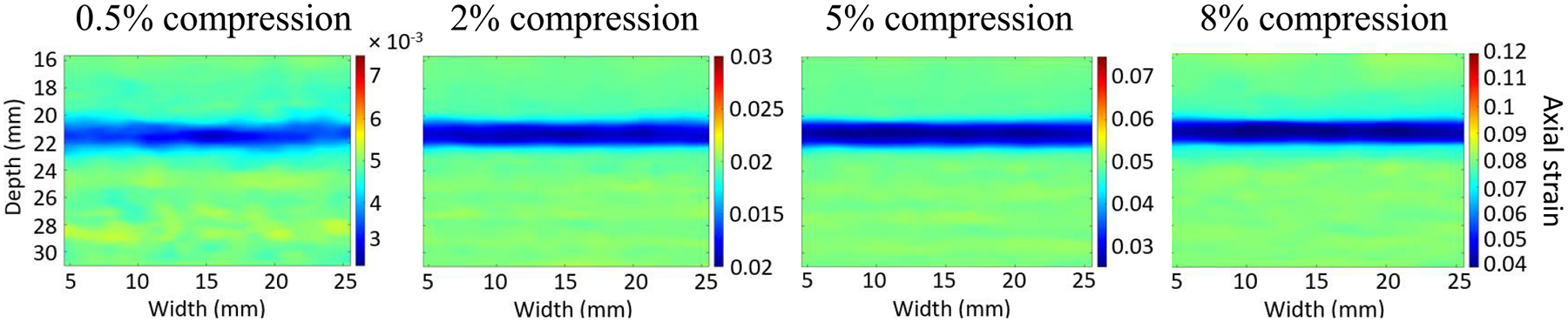
Axial strain results obtained from the thin-layer-inclusion simulated phantom with inhomogeneous Young’s moduli.

Fig. 2 shows the L1-MixTURE axial strain images obtained from the 1.8-mm-thin-layer-inclusion simulated phantom. These strain images are qualitatively acceptable (i.e., smooth background and clearly visible inclusion) at the four investigated compression levels (0.5%, 2%, 5%, and 8%). Although the background smoothness, target–background contrast, and clarity of the inclusion with compression levels of 2%, 5%, and 8% are better when compared with 0.5% compression, these results validate the potential of L1-MixTURE to support a wide range of compression levels.

### B. Validation With Inhomogeneous Poisson’s Ratio

**Fig. 3.**
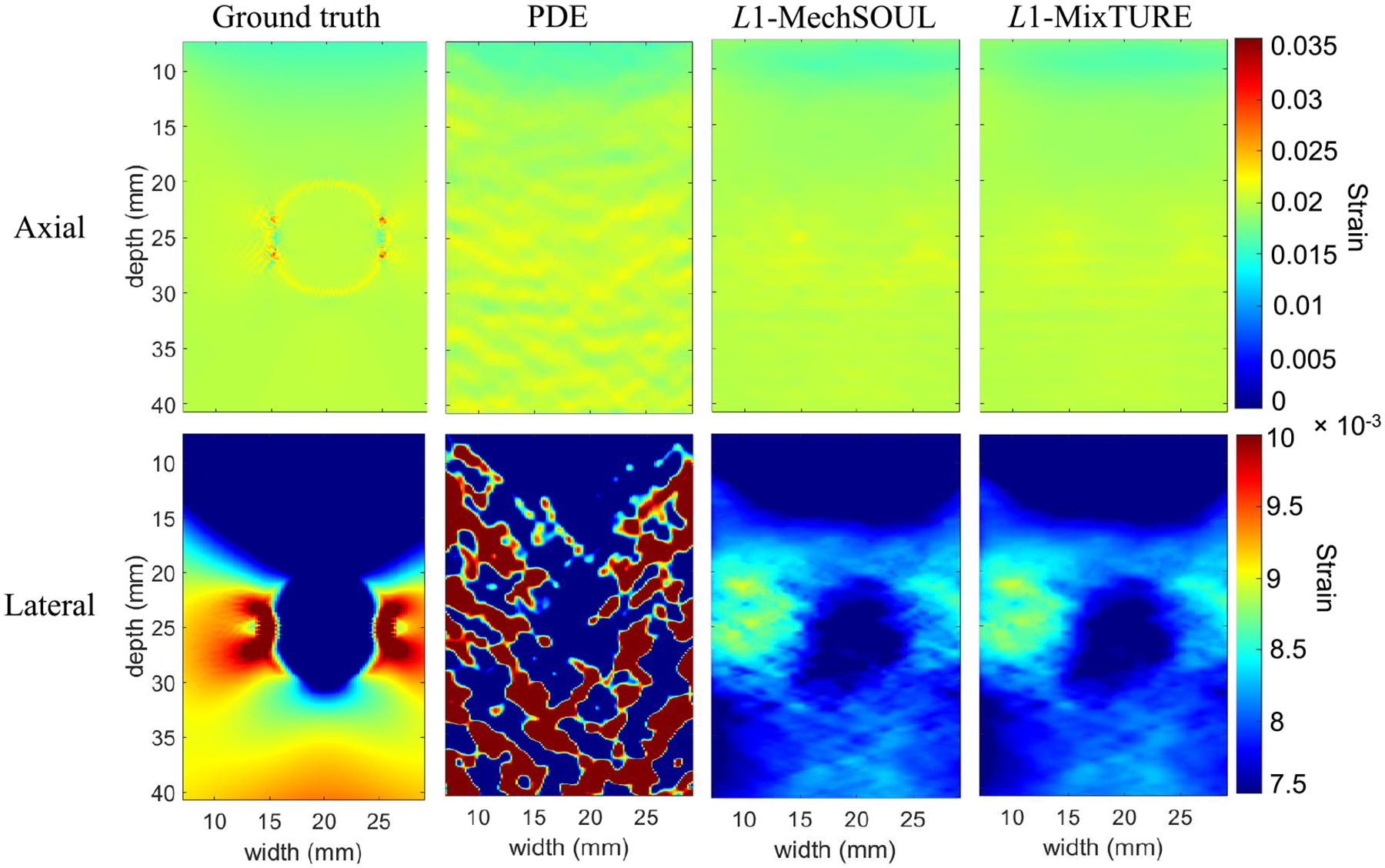
Strain results obtained from the simulated phantom with homogeneous Young’s moduli and inhomogeneous Poisson’s ratios.

Fig. 3 shows axial and lateral strain estimates and the corresponding ground truths for the simulated phantom with different target and background Poisson’s ratios. The ground-truth axial strain is similar throughout the image, as the target and background have the same Young’s modulus. However, an inclusion is revealed in the ground-truth lateral strain image, due to the differences in Poisson’s ratios. Although the PDE approach estimates a noisy but qualitatively acceptable axial strain image, the corresponding lateral strain image is poor. L1-MechSOUL and L1-MixTURE obtain good-quality axial and lateral strain images, with the L1-MixTURE strain images containing less noise than the L1-MechSOUL strain images. This dataset is generally challenging because the effective Poisson’s ratio-based mechanical constraint does not directly improve lateral strain estimates, as the target and background Poisson’s ratios differ. In addition, while the final effective Poisson’s ratio map deviates from the constant initialization by up to 44.25%, it also differs from the corresponding ground truth by up to 149.69%. Considering these challenges (which are responsible for the lateral strain map differences compared with the ground truth), the lateral strain images produced by L1-MechSOUL and L1-MixTURE nonetheless visualize the inclusion. Overall, these results validate the performance of L1-MixTURE when Poisson’s ratios vary within an imaging field of view.

### C. Public Single-Inclusion Simulated Phantom

**Fig. 4.**
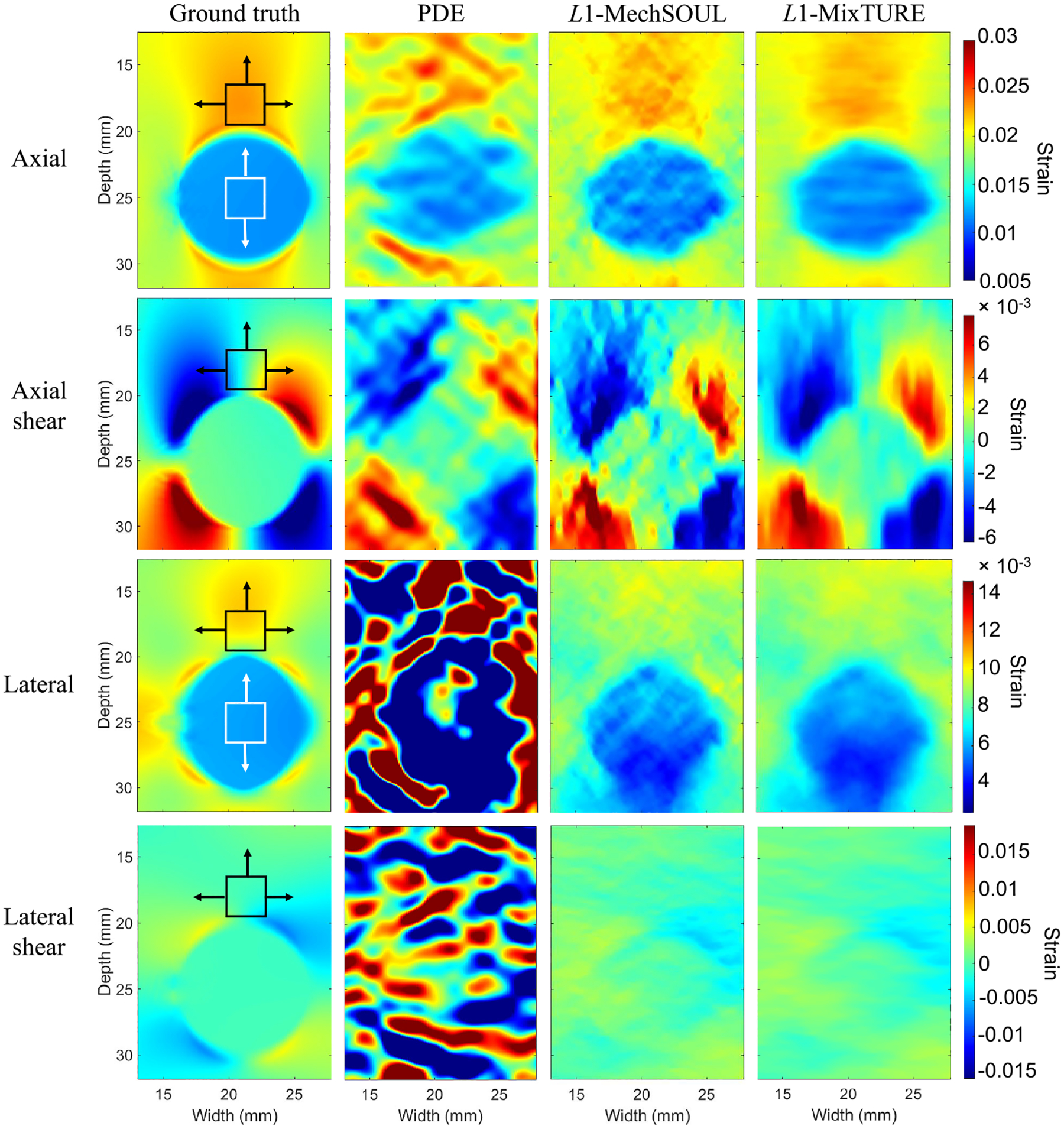
Axial, lateral, axial shear, and lateral shear strain results obtained from the public single-inclusion simulated phantom. The white and black boxes indicate target and background ROIs, respectively, used to calculate one of the 30 SNR measurements (black ROI only) and one of the 120 CNR measurements per indicated strain image. The arrows indicate the directions of ROI displacement to obtain the remaining SNR and CNR values. PDE and L1-MechSOUL are challenged by the Gaussian noise added to the beamformed RF data.

Fig. 4 shows the axial, lateral, axial shear, and lateral shear strains estimated by PDE, L1-MechSOUL, and L1-MixTURE, relative to the ground truth for the simulated single-inclusion phantom. The axial strain and axial shear strain images are generally similar to the ground truth with the three techniques. Although PDE produces sharp inclusion boundaries in the axial strain and axial shear strain images, the structure surrounding the inclusion border is also distorted. L1-MechSOUL axial strain and axial shear strain images contain visible noise.

L1-MixTURE produces axial strain and axial shear strain images that are most similar to the ground truth and visually smoother than the corresponding L1-MechSOUL and PDE results in Fig. 4. However, L1-MixTURE slightly blurs the inclusion edges relative to PDE and L1-MechSOUL as a byproduct of the proposed mixed-derivative-based regularization. The target-to-background contrast and inclusion edges are also most pronounced in the L1-MixTURE axial strain image relative to PDE and L1-MechSOUL. When estimating lateral strain and lateral shear strain images, PDE generally fails, and L1-MixTURE is most similar to the ground truth. In addition, the visual contrast of the lateral strain image produced with L1-MixTURE is higher than that produced by L1-MechSOUL, while the corresponding lateral shear strain results are similar.

Tables II and III report quantitative metrics that support the qualitative observations noted above. When evaluating the axial strain results from the simulated single-inclusion phantom, the MAE, MSSIM, SNR, and CNR improved by 10.61%, 22.06%, 34.23%, and 37.96%, respectively, with L1-MixTURE compared with L1-MechSOUL. Similar improvements (i.e., ranging 0%−28.57%) were observed when evaluating the lateral strain and axial shear strain results, whereas up to 9.36% lateral shear strain improvements were observed when comparing L1-MixTURE to L1-MechSOUL. Percent improvements with L1-MixTURE relative to PDE are not reported because it was previously shown that L1-MechSOUL outperforms PDE [38], and thus our focus is percent improvements of L1-MixTURE relative to L1-MechSOUL.

### D. Multi-Inclusion Simulated Phantom

**Fig. 5.**
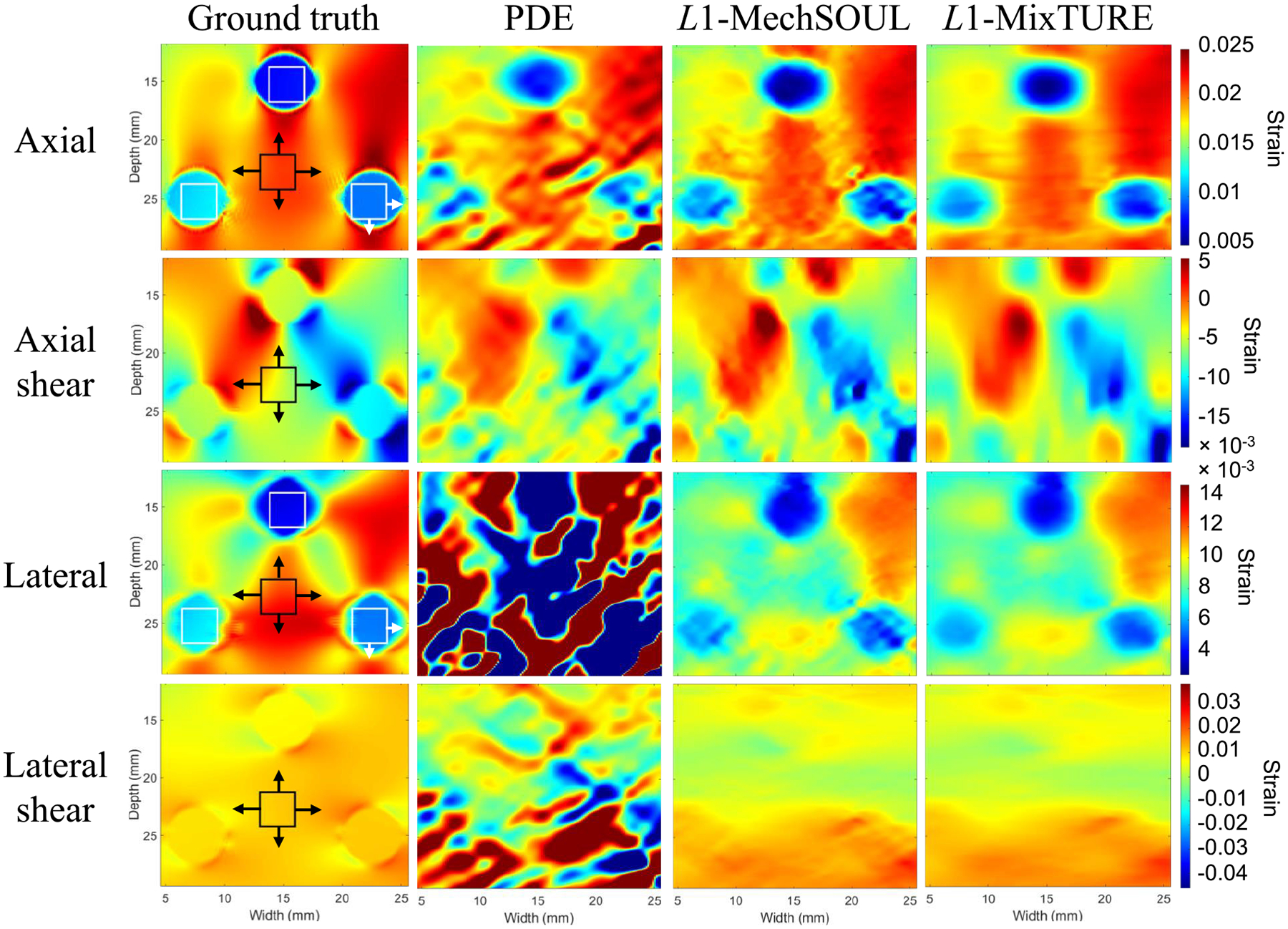
Multi-inclusion simulated phantom axial, lateral, axial shear, and lateral shear strain results. The white and black boxes indicate three target ROIs and one background ROI, respectively, used to calculate one of the 30 SNR measurements (black ROI only) and three of the 120 CNR measurements per indicated strain image. The arrows indicate the directions of ROI displacement to obtain the remaining SNR and CNR values. PDE and L1-MechSOUL are challenged by the simulated surface traction force-induced motion.

Fig. 5 shows the four strain images and the corresponding ground truth obtained with the simulated multi-inclusion phantom. Qualitatively, PDE and L1-MechSOUL produce noisy axial strain and axial shear strain images. In addition, deeper inclusions have poor contrast with the background in the PDE axial strain image and unwanted strain variations in the L1-MechSOUL axial strain image. L1-MixTURE produces smoother axial strain and axial shear strain images that resemble the ground truth, with good target-to-background contrast and sharp edges in the L1-MixTURE axial strain image. When qualitatively assessing the lateral strain images, PDE fails, whereas L1-MechSOUL and L1-MixTURE produce high-quality estimates, with the greatest smoothness, contrast, and similarity to the ground truth achieved with L1-MixTURE. When assessing the lateral shear strain images, L1-MechSOUL and L1-MixTURE outperform PDE in terms of similarity to the ground truth and smoothness, with no qualitative difference between L1-MechSOUL and L1-MixTURE lateral shear strain images.

The quantitative metrics reported in Tables II and III support the qualitative observations noted above. In particular, L1-MixTURE offers improvements ranging from 0% (MSSIM and MAE of lateral shear strain images, MAE of axial and lateral strain images) to 10.33% (SNR of axial strain images) relative to L1-MechSOUL.

### E. Single-Inclusion Experimental Breast Phantom

**Fig. 6.**
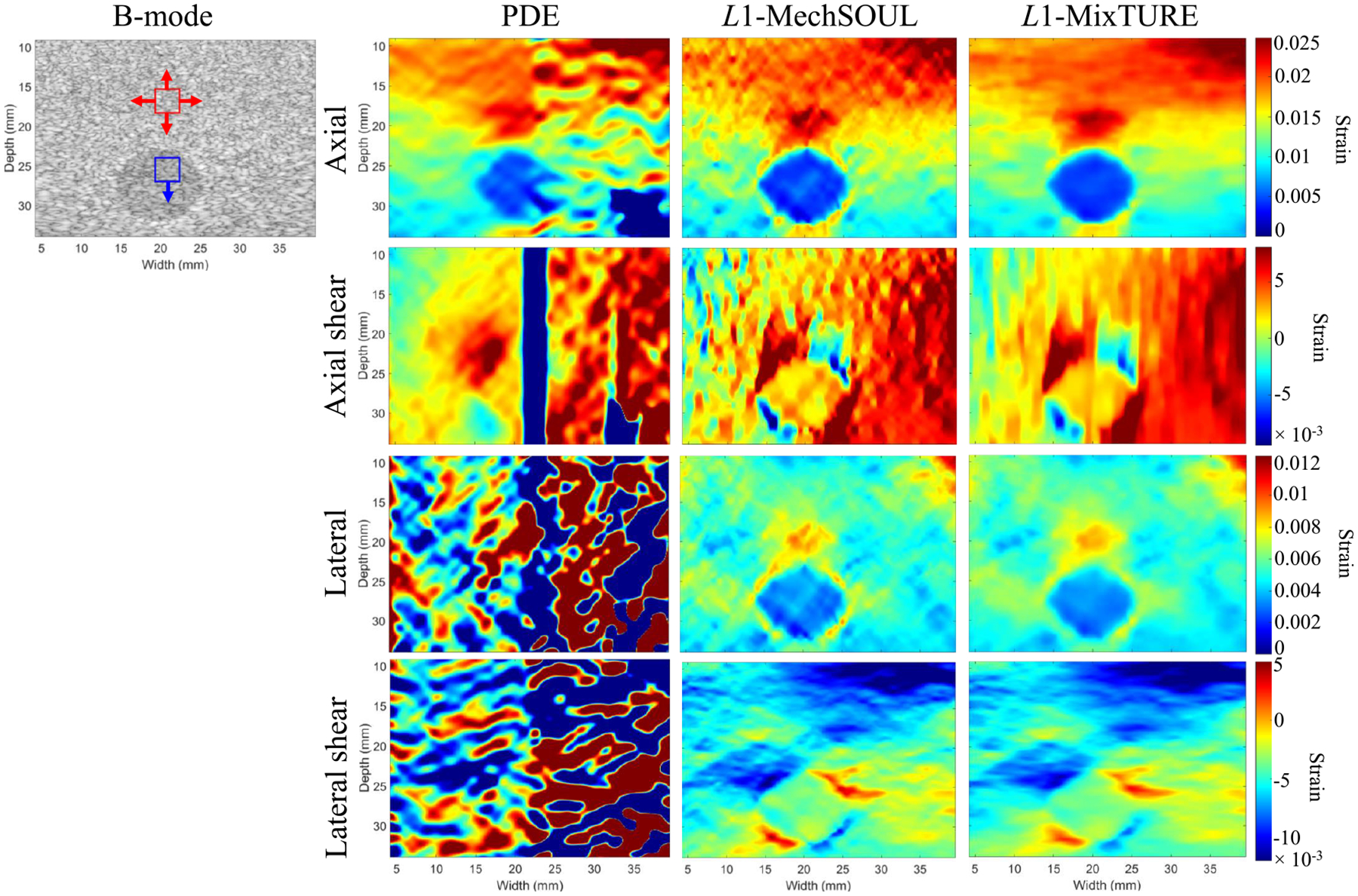
Single-inclusion experimental breast phantom axial, lateral, axial shear, and lateral shear strain results. The blue and red boxes indicate one target ROI and one background ROI, respectively, used to calculate one of the 30 SNR (red ROI only) and one of the 120 CNR measurements associated with these strain images. The arrows indicate the directions of ROI displacement to obtain the remaining SNR and CNR values. The B-mode image is shown with 50-dB dynamic range.

Fig. 6 shows the four strain images and corresponding precompressed B-mode image obtained with the single-inclusion experimental phantom data. PDE obtains an acceptable axial strain image. However, the PDE axial strain image presents a distorted appearance of the inclusion and contains artifacts in the bottom right corner. In addition, PDE axial shear strain images contain vertical artifacts (likely caused by the high level of compression applied to this phantom), and the lateral strain and lateral shear strain images produced with PDE are unacceptable. L1-MechSOUL and L1-MixTURE produce good-quality axial, axial shear, lateral, and lateral shear strain images. L1-MixTURE reduces noise that appears in the L1-MechSOUL images and provides the smoothest strain images without sacrificing boundary sharpness and target-to-background contrast.

The quantitative values of SNR and CNR reported in Table III substantiate the qualitative findings mentioned above. L1-MixTURE improved the mean SNR and CNR of the axial strain images by 56.41% and 59.23%, respectively, and improved the mean SNR and CNR of the lateral strain images by 23.38% and 30.28%, respectively, over L1-MechSOUL. L1-MixTURE improved the mean SNR of the axial and lateral shear strains improved by 67.82% and 14.44%, respectively, over L1-MechSOUL.

### F. Multi-Inclusion Experimental Breast Phantom

**Fig. 7.**
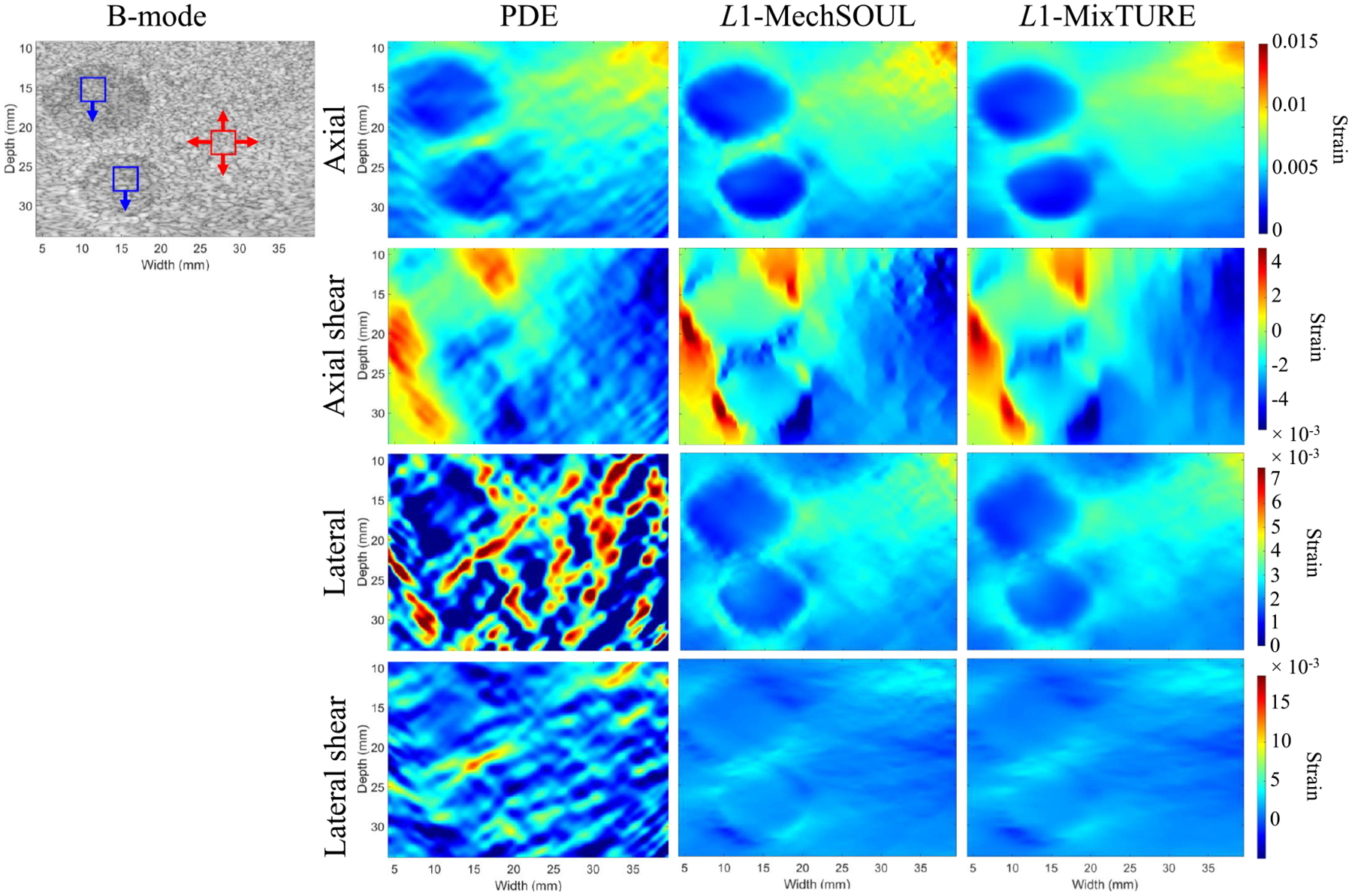
Multi-inclusion experimental breast phantom axial, lateral, axial shear, and lateral shear strain results. The blue and red boxes indicate two target ROIs and one background ROI, respectively, used to calculate one of the 30 SNR (red ROI only) and two of the 120 CNR measurements associated with these strain images. The arrows indicate the directions of ROI displacement to obtain the remaining SNR and CNR values. The B-mode image is shown with 50-dB dynamic range.

Fig. 7 shows the four strain images and corresponding precompressed B-mode image obtained with the multi-inclusion experimental phantom data. PDE, L1-MechSOUL, and L1-MixTURE each produce good-quality axial strain and axial shear strain images. However, PDE does not display the right border of the deeper inclusion in the axial strain image. In addition, PDE and L1-MechSOUL have a noisy background in the axial strain and axial shear strain images, and L1-MechSOUL has visible noise surrounding the inclusion boundaries of the axial shear strain image. These noisy features appear smoother with sharper edges in the L1-MixTURE axial strain and axial shear strain images. The target-to-background contrast is additionally excellent in the L1-MixTURE axial strain image. The lateral strain image produced with PDE is generally uninterpretable, although the shallower lesion is visualized. Better lateral strain images are obtained with L1-MechSOUL and L1-MixTURE, and the smoothest background and target visibility in the lateral strain images are achieved with L1-MixTURE. The noisiest lateral shear strain results were obtained with PDE, whereas L1-MixTURE renders a marginally smoother lateral shear strain than L1-MechSOUL.

The SNR and CNR values reported in Table III are consistent with the qualitative observations noted above. The mean SNR and CNR of the axial strain images improved by 10.87% and 9.21%, respectively, with L1-MixTURE relative to L1-MechSOUL. The mean SNR and CNR of the lateral strain images improved by 16.91% and 12.79%, respectively, with L1-MixTURE relative to L1-MechSOUL. The mean SNR of the axial and lateral shear strains improved by 31.07% and 7.04%, respectively, with L1-MixTURE relative to L1-MechSOUL.

### G. In Vivo Breast Mass

**Fig. 8.**
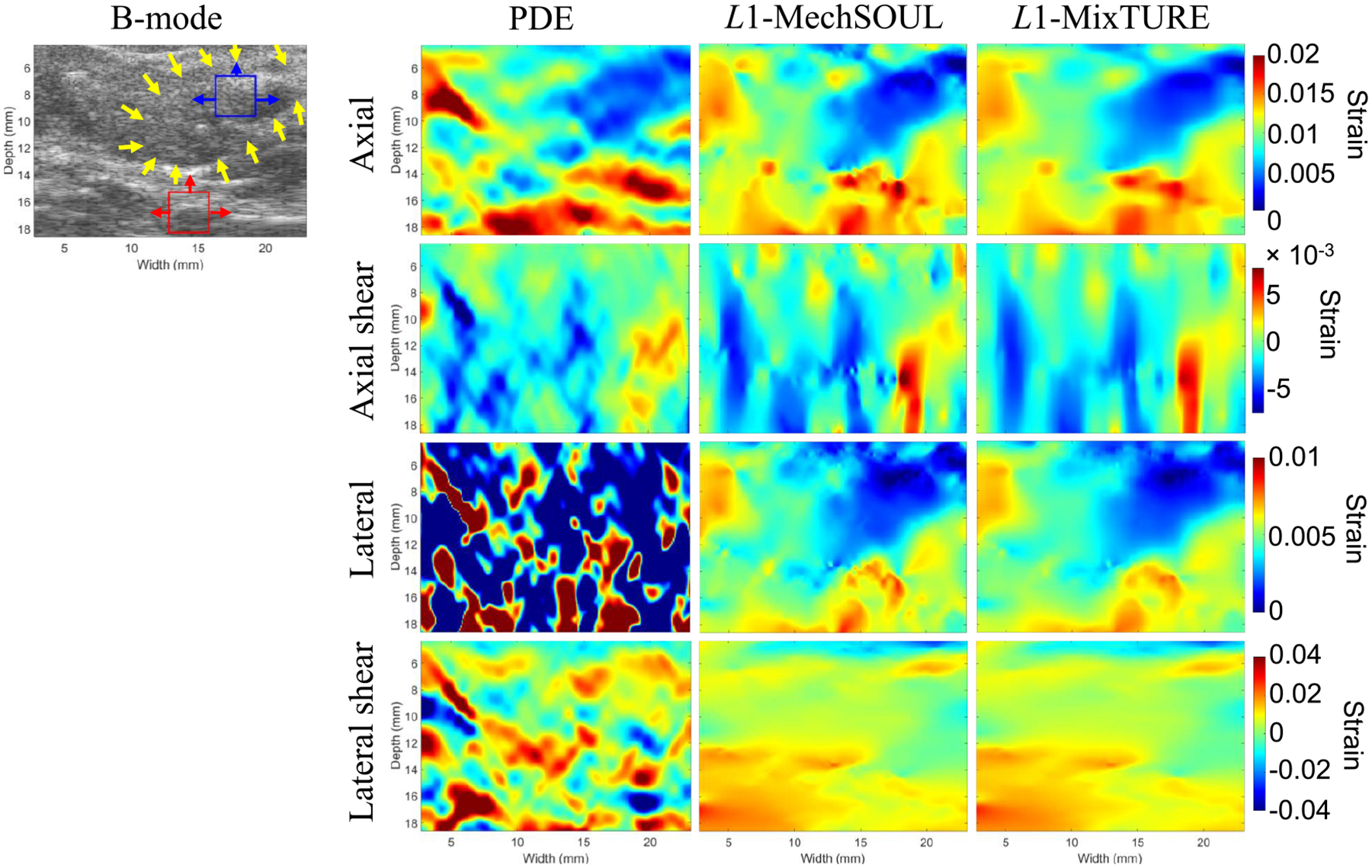
In vivo breast mass axial, lateral, axial shear, and lateral shear strain results. The blue and red boxes indicate target and background ROIs, respectively, used to calculate one of the 30 SNR and 120 CNR measurements associated with these strain images, with connected arrows indicating the directions of ROI displacement to obtain the remaining SNR and CNR values. The yellow arrows delineate the breast mass. The B-mode image is shown with 50-dB dynamic range.

Fig. 8 shows the four strain images and corresponding precompressed B-mode image obtained from the in vivo breast mass. An acceptable axial strain image is produced by PDE, although it is the noisiest among the three techniques compared. L1-MechSOUL axial strain image is qualitatively better than that produced by PDE, although there is unexpected variability in the background and surrounding the benign mass. L1-MixTURE reduces this estimation variability, while maintaining sharp edges and high target-to-background contrast. The axial and lateral shear strain images are noisiest with PDE, which also produces uninterpretable lateral strain images. L1-MechSOUL produces results with less noise than that in the corresponding axial shear, lateral, and lateral shear strain images produced with PDE. L1-MixTURE further reduces the noise in these three strain images. In general, the strain images reconstructed from this in vivo breast dataset are worse than the strain images pertaining to the simulated and phantom datasets because of noise from various sources, such as unwanted motion from vessel pulsations and unexpected patient motion.

The quantitative results in Table III support the qualitative observations noted above. L1-MixTURE improved the mean SNR and CNR of the axial strain images by 23.71% and 19.92%, respectively, and improved the mean SNR and CNR of the lateral strain images by 25.53% and 14.64%, respectively, when compared with the corresponding results produced by L1-MechSOUL. In addition, L1-MixTURE improved the mean SNR of the axial and lateral shear strain images by 6.63% and 7.01%, respectively, relative to the corresponding results produced by L1-MechSOUL.

### H. Execution Times

The execution times for MATLAB implementations of PDE, L1-MechSOUL, and L1-MixTURE on a sixth-generation Intel Core-i5 CPU with 32-GB RAM are 511.79, 30.48, and 36.86 s, respectively, when calculating the displacement fields between two 2000 samples × 200 samples ultrasound RF frames.

## IV. Discussion

This work is the first to present a general L1-norm-based displacement tracking technique that combines mixed and unmixed second derivatives with a mechanical relationship driven by the effective Poisson’s ratio between the axial and lateral strain components, ensuring a sharp and accurate estimation of the total strain tensor. Qualitative and quantitative assessments with simulated, phantom, and in vivo breast data demonstrate the superiority of the proposed L1-MixTURE technique. The qualitative results (see Figs. 4–8) indicate that L1-MixTURE produces smoother axial, lateral, axial shear, and lateral shear strains, while maintaining sharp edges. The error- and similarity-related quantitative metrics, namely, MAE and MSSIM (see Table II), demonstrate that L1-MixTURE reduces the estimation error and produces strain images similar to the ground-truth images. The improved SNR and CNR demonstrate that L1-MixTURE suppresses noise, while preserving contrast, relative to the benchmarked competing approaches (see Table III). Collectively, these quantitative metrics demonstrate that L1-MixTURE provides accurate information about elastic contrast, which is expected to improve diagnoses of pathologic tissue [75].

Overall, the most important attribute of L1-MixTURE is that it facilitates high-quality estimations of four strain components (axial, lateral, axial shear, and lateral shear). This finding highlights the strength of the combination of L1-norm, a mechanical constraint driven by the effective Poisson’s ratio, and mixed and unmixed second-order continuity constraints. As window-based methods are commonly used to compare new tracking techniques, we additionally demonstrated that L1-MixTURE qualitatively and quantitatively outperforms PDE.

Similar to [38], we adopt a cross-validation strategy to optimize the parameter set pertaining to each technique. Optimal parameter sets, determined after tuning with validation data, were directly used to generate the results presented in this manuscript, demonstrating the robustness of our parameter-tuning. In particular, the parameter sets were optimized on different frames than the test frames, although the parameter sets were optimized individually for simulated, phantom, and in vivo datasets because of the differences in mechanical properties and noise distributions inherent in each dataset. However, it is promising that the same parameter values were used for four simulated phantoms (see Table I). It is additionally promising that the same values were used when implementing both L1-MechSOUL and L1-MixTURE, with the exception of the additional tuning required for the newly added mixed derivative-related parameters to implement L1-MixTURE. In addition, as shown in previous work [16], [32], the performance of an energy-based technique is robust to parameter value alterations as large as 50%.

Ultrasound displacement tracking is often known to be more accurate in the axial direction than the lateral direction for the following three reasons: 1) the point-spread function is narrower in the axial direction; 2) there is an echo carrier frequency in the axial direction that does not exist in the lateral direction; and 3) the sampling rate is higher in the axial direction than the lateral direction. Therefore, the axial and axial shear strains (see Figs. 4–8) generally have better quality than the lateral and lateral shear strains, as they are obtained from the axial displacement field. In addition, L1-MechSOUL and L1-MixTURE exploit the effective Poisson’s ratio-based relation between axial and lateral strains to ensure good-quality lateral strain images. However, lateral shear strain is calculated from the lateral displacement field and is not complemented by any mechanical constraint. Therefore, the lateral shear strain quality is worse when compared with the axial, axial shear, and lateral strain components.

One unique feature observed in Fig. 4 is the relatively high estimation errors below the inclusion in L1-MechSOUL and L1-MixTURE images. A possible explanation is high signal decorrelation in deep tissue regions due to large displacement magnitude. However, this depth-dependent estimation error variation was not observed in any other experiment.

From a clinical perspective, the reduced noise, preserved edges, high strain CNR, and improved shear strain estimates with L1-MixTURE are positive attributes for pathologic tissue identification (e.g., breast mass diagnosis). Noisy strain images present ambiguous information and increase the risk of diagnostic error. Therefore, the high-SNR axial and lateral strain images obtained by L1-MixTURE demonstrate its potential to advance low-variance, high-confidence, noninvasive pathology delineation. In addition, the edge-preserving attribute of L1-MixTURE indicates its ability to image small-sized (e.g., ≤2-mm diameter) lesions, which is supported by visualization of the 1.8-mm inclusion in Fig. 2. The high axial and lateral strain CNR (see Table III) reveals that L1-MixTURE is capable of capturing low elastic contrast between healthy and pathologic tissues (i.e., low difference between the elasticity of two tissue types). The combined ability to image small-sized, low-elastic-contrast targets suggests that L1-MixTURE has the potential to benefit early cancer detection and treatment monitoring. Shear strain was previously implemented as a biomarker for tumor malignancy (particularly, both the area of the shear region, defined as a region with alternating positive and negative shear strains, and the proximity between the shear region and lesion were reported as features of interest [47]). Therefore, improved shear strain estimation reported in Tables II and III suggests the potential of L1-MixTURE to assist with tumor classification with high accuracy. In addition, it is promising that L1-MixTURE shows the shear regions more clearly in Figs. 4–8, relative to that produced by PDE and L1-MechSOUL for the same targets.

One limitation of our study is that the level of mechanical compression can alter the elasticity of underlying tissues [76], [77], [78], [79], and we did not control this variable during the experimental or in vivo data acquisitions, as our focus is strain imaging rather than providing quantitative elastic properties. In addition, a technical limitation is that both L1-MechSOUL and L1-MixTURE approximate the L1-norm with a differentiable substitute to facilitate the simultaneous optimizations of data and regularization terms. This strategy trades the maximum potential of L1-norm regularization with the ease of optimization. Future work will address this limitation by incorporating a sophisticated optimization technique, such as the alternating direction method of multipliers, which is capable of optimizing different components of the cost function individually, requiring no inexact approximation for the L1-norm [80], [81], [82]. Future work will additionally develop a theoretical framework to calculate the optimal strain range for L1-MixTURE, possibly similar to a previously reported strain filter approach [74].

## V. Conclusion

We present a novel L1-norm-based second-order regularizer in a mechanically inspired total strain tensor imaging framework named L1-MixTURE. The proposed approach exploits both mixed and unmixed displacement derivatives to formulate the second-order regularization scheme. L1-MixTURE produces smooth axial, lateral, axial shear, and lateral shear strain maps while preserving or enhancing edge clarity and contrast. Relative to L1-MechSOUL (i.e., the precursory displacement tracking technique to L1-MixTURE), L1-MixTURE achieved strain image quality improvements as large as 25.53%−67.82% with single-and multi-inclusion simulated and experimental breast phantom datasets and when imaging an in vivo breast mass. These qualitative and quantitative improvements have the potential to translate to large gains with regard to improved techniques for breast ultrasound (and other clinical applications), considering the millions of patients worldwide who annually undergo diagnostic and interventional ultrasound imaging.

## Figures and Tables

**TABLE I:** L1-MechSOUL and L1-MixTURE Parameters

	Simulated data	Phantom data	*In vivo* data
$\alpha_{1s}$	150	120	20
$\alpha_{2s}$	0.2	0.4	0.05
$\alpha_{3s}$	0.045	0.036	0.006
$\alpha_{\text{mix}}$	56.25	45	7.5
$\beta_{1s}$	$2\alpha_{1s}/3$	$2\alpha_{1s}/3$	$2\alpha_{1s}/3$
$\beta_{2s}$	$\alpha_{2s}/4$	$\alpha_{2s}/4$	$\alpha_{2s}/4$
$\beta_{\text{mix}}$	$\alpha_{\text{mix}}/2$	$\alpha_{\text{mix}}/2$	$\alpha_{\text{mix}}/2$
$w_{f}$	0.001	0.0006	0.0006
$w_{s}$	0.025	0.01	0.01

**TABLE II:** MSSIM and MAE Achieved With the Single-Inclusion and Multi-Inclusion Simulated Phantoms. Bold Text Indicates the Best Results per Metric per Strain Component Direction per Dataset

	MSSIM				MAE			
	Axial	Axial Shear	Lateral	Lateral Shear	Axial	Axial Shear	Lateral	Lateral Shear
**Single-Inclusion**								
PDE	0.52	0.55	0.0021	0.04	0.0015	0.0011	0.0084	0.0117
L1-MechSOUL	0.68	0.62	0.63	**0.53**	0.00066	0.00071	**0.0011**	**0.0014**
L1-MixTURE	**0.83**	**0.73**	**0.72**	**0.53**	**0.00059**	**0.00053**	**0.0011**	**0.0014**
**Multi-Inclusion**								
PDE	0.71	0.71	0.06	0.12	0.0018	0.0016	0.014	0.018
L1-MechSOUL	0.83	0.81	0.83	**0.69**	**0.001**	0.00086	**0.001**	**0.005**
L1-MixTURE	**0.86**	**0.84**	**0.86**	**0.69**	**0.001**	**0.00082**	**0.001**	**0.005**

**TABLE III:** SNR and CNR Values (Mean ± Standard Deviation) Achieved With the Simulated, Experimental Phantom, and In Vivo Data. Bold Text indicates the Best Results per Metric per Strain Component Direction per Dataset

	SNR				CNR	
	Axial	Axial Shear	Lateral	Lateral Shear	Axial	Lateral
**Single-Inclusion Simulated Phantom**						
PDE	16.22 ± 3.97	2.02 ± 0.81	1.49 ± 0.35	1.55 ± 0.23	8.60 ± 1.79	1.14 ± 0.74
L1-MechSOUL	39.47 ± 8.84	2.24 ± 0.77	35.22 ± 5.23	1.71 ± 0.37	17.78 ± 2.50	10.43 ± 1.34
L1-MixTURE	**52.98** ± **17.69**	**2.88** ± **1.29**	**42.26** ± **10.52**	**1.87** ± **0.49**	**24.53** ± **5.92**	**11.97** ± **2.02**
**Multi-Inclusion Simulated Phantom**						
PDE	24.45 ± 8.32	5.38 ± 2.48	2.27 ± 0.89	1.50 ± 0.35	4.73 ± 2.83	2.07 ±1.19
L1-MechSOUL	45.29 ± 18.31	6.11 ± 4.65	35.91 ± 20.23	4.77 ± 4.36	11.30 ± 3.14	9.89 ± 4.45
L1-MixTURE	**49.97** ± **20.81**	**6.36** ± **4.71**	**37.95** ± **22.05**	**4.86** ± **4.58**	**11.89** ± **3.27**	**10.61** ± **4.69**
**Single-Inclusion Breast Phantom**						
PDE	18.35 ± 12.44	1.80 ± 1.10	1.89 ± 0.87	1.87 ± 0.51	10.97 ± 6.09	1.02 ± 0.83
L1-MechSOUL	16.15 ± 5.46	2.89 ± 1.92	12.49 ± 3.42	7.41 ± 2.65	13.32 ± 4.59	5.68 ± 2.32
L1-MixTURE	**25.26** ± **11.52**	**4.85** ± **3.48**	**15.41** ± **4.97**	**8.48** ± **2.91**	**21.21** ± **9.65**	**7.40** ± **3.51**
**Multi-Inclusion Breast Phantom**						
PDE	16.12 ± 5.87	8.31 ± 3.33	1.61 ± 0.49	1.52 ± 0.45	9.71 ± 4.09	0.88 ± 0.66
L1-MechSOUL	25.03 ± 9.32	12.52 ± 6.84	25.55 ± 13.73	5.54 ± 3.64	13.35 ± 5.12	9.77 ± 5.07
L1-MixTURE	**27.75** ± **10.26**	**16.41** ± **9.46**	**29.87** ± **16.99**	**5.93** ± **4.01**	**14.58** ± **5.60**	**11.02** ± **5.92**
***In Vivo* Mass**						
PDE	5.49 ± 2.27	1.85 ± 0.72	1.45 ± 0.20	1.38 ± 0.22	4.35 ± 1.54	0.72 ± 0.53
L1-MechSOUL	13.41 ± 5.92	1.96 ± 1.12	12.73 ± 5.52	4.85 ± 2.75	7.28 ± 2.73	6.49 ± 1.92
L1-MixTURE	**16.59** ± **7.66**	**2.09** ± **1.13**	**15.98** ± **7.76**	**5.19** ± **3.17**	**8.73** ± **3.35**	**7.44** ± **2.12**

## Highlights

- We formulated, optimized, and validated a novel L1-norm-based second-order regularizer for ultrasound displacement tracking that penalizes both mixed and unmixed second-order displacement derivatives.
- The proposed technique (i.e., L1-MixTURE) produces normal and shear strain images that outperform two previous techniques when applied to simulated, experimental, and in vivo breast datasets.
- Results demonstrate the potential of L1-MixTURE to advance the state-of-the-art in elastography-guided diagnostic and interventional decisions.
